# High circulating endocan in chronic kidney disease? A systematic review and meta-analysis

**DOI:** 10.1371/journal.pone.0289710

**Published:** 2023-08-09

**Authors:** Amirmohammad Khalaji, Amir Hossein Behnoush, Yasmin Mohtasham Kia, Parsa Alehossein, Pegah Bahiraie

**Affiliations:** 1 School of Medicine, Tehran University of Medical Sciences, Tehran, Iran; 2 Endocrinology and Metabolism Population Sciences Institute, Non-Communicable Diseases Research Center, Tehran University of Medical Sciences, Tehran, Iran; 3 School of Medicine, Iran University of Medical Sciences, Tehran, Iran; 4 Neuroscience Research Center, Shahid Beheshti University of Medical Sciences, Tehran, Iran; 5 School of Medicine, Shahid Beheshti University of Medical Sciences, Tehran, Iran; University of Verona: Universita degli Studi di Verona, ITALY

## Abstract

**Background:**

Chronic kidney disease (CKD) is one of the leading causes of morbidity and mortality worldwide. Endothelial dysfunction has been suggested to be involved in the pathophysiology of CKD. Endocan, as an endothelial factor, has been shown to increase in several diseases. The current systematic review and meta-analysis was performed with the aim of determining the association between endocan levels and CKD.

**Methods:**

Four international databases, including PubMed, Embase, Scopus, and Web of Science were searched for relevant studies. Afterward, screening and extraction of data were performed. We conducted a random-effect meta-analysis to calculate the standardized mean difference (SMD) and 95% confidence interval (CI) to compare circulating endocan levels between patients with CKD (including patients undergoing hemodialysis) and healthy controls. Subgroup analysis based on the specimen in which endocan was measured (serum or plasma) was also performed.

**Results:**

After screening by title/abstract and full-text review by the authors, 20 studies were included. Meta-analysis revealed that serum endocan is higher in CKD patients compared to healthy controls (SMD 1.34, 95% CI 0.20 to 2.48, p-value<0.01). This higher endocan level was also observed in the subgroup of studies that measured plasma endocan while this was not the case for the subgroup of studies assessing serum endocan. Meta-analysis was also performed for comparison of CKD patients without other comorbidities and healthy controls, which resulted in the same conclusion of higher endocan levels in patients with CKD (SMD 0.74, 95% CI 0.52 to 0.95, p-value<0.01). Moreover, endocan was associated with cardiovascular diseases in CKD.

**Conclusion:**

Our study demonstrated that endocan is significantly increased in patients with CKD. This can have clinical implications as well as highlight the need for future research investigating the diagnostic and prognostic role of endocan in CKD.

## 1. Introduction

In kidney and lung endothelial cells, where its expression is controlled by cytokines, the endothelial cell-specific molecule (ESM)-1 was first discovered to be expressed. This molecule was originally known as ESM-1 because of its extremely limited spread to vascular endothelial cells. Further investigation established that ESM-1 is a member of the proteoglycans family and it was called endocan. Vascular endothelial growth factors (VEGF)-A, VEGF-C, interleukin (IL)-1, tumor necrosis factor (TNF), transforming growth factor-1, and fibroblast growth factor (FGF)-2 up-regulate endocan production, whereas phosphatidyl-inositide3-kinases (PI3K) and interferon-gamma down-regulate it. ESM-1 blocks the interaction of soluble intercellular adhesion molecule (ICAM)-1, an endothelial cell adhesion protein, with CD11a/CD18 binding sites on the cell surface of lymphocytes and monocytes by directly binding to the integrin CD11a/CD18 on these cells. These characteristics imply that ESM-1 may control cellular invasion during inflammatory processes. Additionally, because the endocan expression is induced by inflammatory mediators, blood levels of this soluble proteoglycan may closely represent the presence and degree of inflammation as well as the effectiveness of the treatment [1,2]. Endocan is overexpressed in conditions like cancer, sepsis, obesity, or inflammatory disease, and it’s linked to patients’ outcomes in those conditions, including sepsis and cancer, according to growing experimental data [3].

Between 8% and 16% of people globally are affected by chronic kidney disease (CKD), which is frequently overlooked by patients and medical professionals. A glomerular filtration rate (GFR) of less than 60 mL/min/1.73 m^2^, albuminuria of at least 30 mg per 24 hours, or signs of kidney damage (such as hematuria or structural abnormalities like polycystic kidneys or dysplastic kidneys) that last for longer than three months are considered to be indicators of kidney damage. As progressive CKD is linked to end-stage kidney disease (ESKD), mortality, cardiovascular diseases, and other negative clinical outcomes, early diagnosis and treatment by primary care physicians is crucial [4]. It is becoming more widely accepted that chronic inflammation plays a role in renal fibrosis and ESKD.

One of the main pathophysiological factors leading to the link between CKD and endothelial dysfunction has been identified, and it may partially account for the intensity of the graded correlation between worsening renal function [5]. Endocan is implicated in inflammation and endothelial dysfunction and may serve as a standalone risk factor for suboptimal clinical outcomes in a variety of diseases [6]. Endocan caused serious vascular inflammatory reactions in mice and human umbilical vein endothelial cells (HUVECs) by compromising the integrity of the vascular barrier. Its blood levels may serve as a new indicator of endothelial cell failure because they are correlated with the patient’s health status and prognosis [7].

It is now crucial to diagnose CKD early and identify those who are most likely to develop ESKD. Existing indicators like proteinuria, estimated GFR (eGFR), and creatinine levels appear to be inadequate. As a result, novel biomarkers, like endocan, are needed to track the development of CKD. In our study, we reviewed several studies that measured the concentration of endocan in CKD patients and discovered that it was greater than the control populations, particularly when patients were in a more advanced state of the disease or had comorbid conditions like diabetes or cardiovascular diseases [6,8–10]. However, some other studies found different findings. For instance, Kosir et al. investigated whether endocan levels in chronic heart failure (HF) patients were comparable in the CKD and non-CKD groups [11].

In this paper, a comprehensive evaluation and meta-analysis of research articles from different databases were conducted to combine data from studies on endocan, addressing the link of this special biomarker with CKD.

## 2. Methods

### 2.1. Search strategy and inclusion criteria

This systematic review and meta-analysis was conducted according to the Preferred Reporting Items for Systematic Reviews and Meta-Analyses (PRISMA) 2020 guidelines [12]. The PRISMA checklist is available in S1 Table in S1 File. A comprehensive literature search was conducted in four international databases, including PubMed, Embase, Scopus, and Web of Science from inception until February 13, 2023. The main keywords comprised all MeSH and non-MeSH terms related to “chronic kidney disease” AND “endocan”. The complete details of the search in each database are shown in S2 Table in S1 File. This study was registered in the PROSPERO registry (CRD42023423593).

We included the studies if the studies evaluated plasma/serum endocan in CKD patients and controls or in different CKD stages. Review studies, meeting abstracts, case reports, and non-English studies were excluded.

### 2.2. Screening and extraction

After the removal of duplicates, two reviewers (AK and AHB) screened the studies based on their titles/abstracts. Then, full-text screening was performed independently by these two authors. Disagreement in each of the stages was resolved by discussion with a third author (PB).

The extraction of studies’ data was based on the following items: 1) first author’s name, 2) location of study, 3) number of individuals in each group and population characteristics, 4) mean age, and male percentage in each study group, 5) main findings of each study and 6) serum/plasma levels of endocan in CKD patients (or each of the stages) and healthy controls

### 2.3. Quality assessment

Two independent reviewers (AHB and AK) evaluated the risk of bias in included studies using the “Newcastle-Ottawa Quality Assessment Scale” (NOS) [13] for observational studies. This scoring system is comprised of three main domains of bias risk including selection, comparability, and outcome. Qualities of “very good”, “good”, “satisfactory”, and “unsatisfactory” were given to the scores of 9–10, 7–8, 5–6, and <5, respectively.

### 2.4. Statistical analysis

All analyses were performed in STATA (version 17, Stata Corp.) with a p-value of <0.05 as the statistical significance threshold. Random-effect meta-analysis (restricted maximum likelihood (REML)) was conducted to calculate the standardized mean difference (SMD) and 95% confidence interval (CI) in comparison of endocan levels between CKD patients and healthy individuals. Where median and interquartile ranges (IQR) were reported for endocan levels, they were converted to mean and standard deviation (SD) using the methods suggested by Luo et al. and Wan et al. [14,15]. Moreover, combining group means and SDs was done using the formulas suggested by the Cochrane Handbook [16].

Higgins’ I-square test based on Cochrane’s *Q* was used to assess the heterogeneity among studies. Low, moderate, and high heterogeneity were for ≤25%, 26–75%, and ≥75%. Subgroup analysis based on the specimen (serum or plasma) and meta-regression based on mean age, publication year, sample size, and male percentage were performed to identify the source of heterogeneity. The outlier studies were identified using the Galbraith plot and analysis was reperformed without them. Sensitivity analysis was also performed with the aim of assessment of the effect of each study on the overall pooled effect size. This was done by removing each of the studies from the analysis and checking if the result was changed in terms of significance. Finally, an assessment of publication bias was done by visual inspection of funnel plots and Begg’s and Egger’s statistical tests [17,18].

## 3. Results

### 3.1. Literature search results

**Fig 1:**
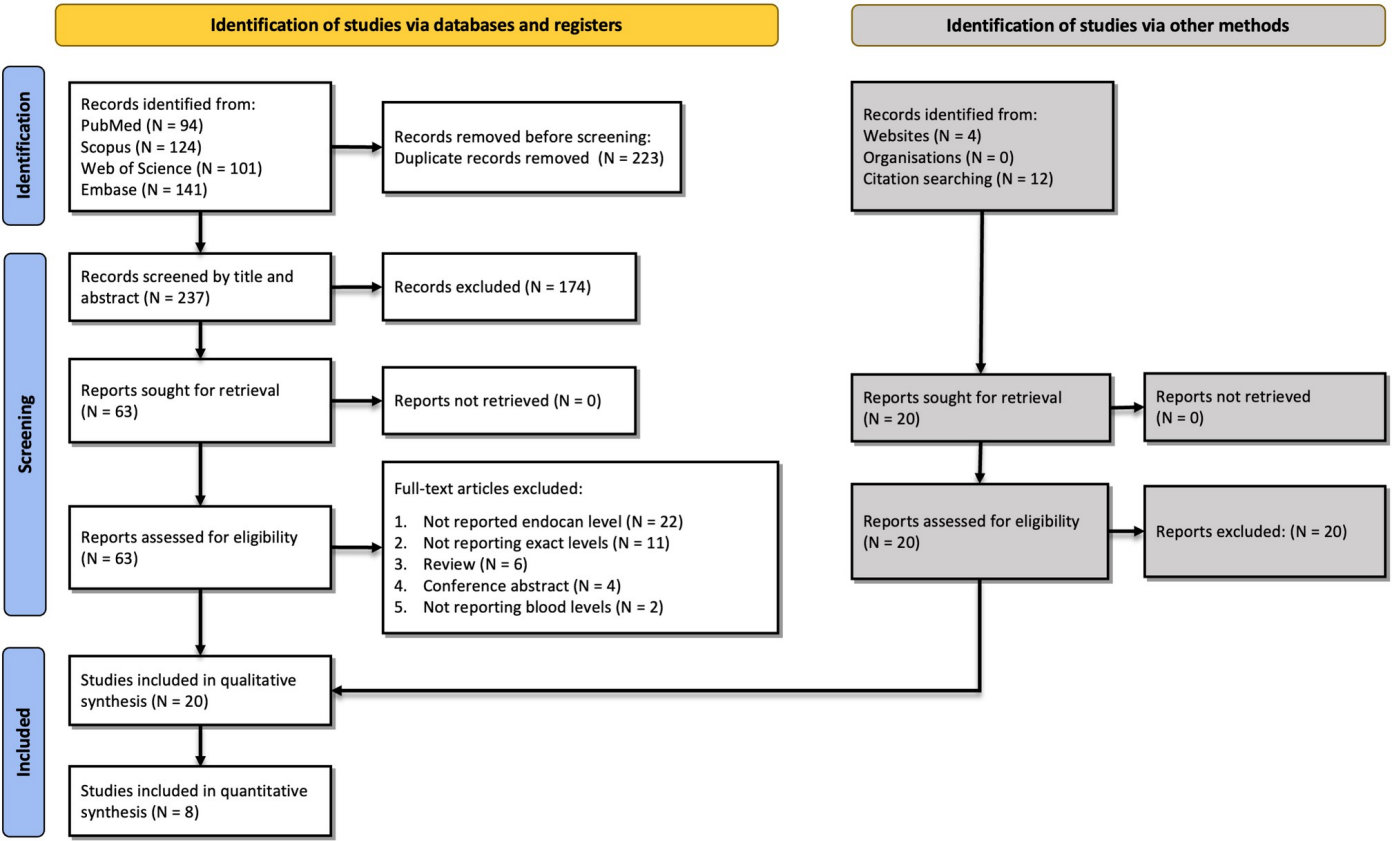
Flow diagram summarizing the selection of eligible studies based on the PRISMA guidelines.

Our primary database search resulted in 460 studies: 94 from PubMed, 124 from Scopus, 141 from Embase, and 101 from Web of Science. After removing duplicates, 237 records remained. Records were screened based on title/abstract, after which 63 studies remained. Based on full-text screening, 20 studies were included. PRISMA flowchart and reasons for exclusion are shown in Fig 1.

### 3.2. Study characteristics

A total of 20 studies were included in our systematic review [3,6,8–11,19–32] and details of them are shown in Table 1. The number of individuals included in these studies is 2316 with a mean age of 53.52 ± 12.38 years while 59.7% of them were male. Turkey [3,19,21], and China [20,30,32] were the leading countries in which studies were conducted. Quality assessment based on NOS showed that all studies had high qualities (Table 2).

**Table 1:** Characteristics of included studies.

Author	Year	Design	Location	Specimen	Population	N total	Age	% Male	Main Findings
Atis et al.	2016	Prospective cohort	Turkey	Serum	Renal transplant patients	38	39.9 ± 11.5	73.7	Endocan levels significantly decreased after treatment with vitamin D in renal transplant recipients (from 621.5 [188–2044] pg/ml to 538 [167–2044] pg/ml, P = 0.001).
Bao et al.	2018	Cross sectional	China	Serum	Non-dialysis stage 5 CKD patients	54	48.2 ± 14.9	59.2	Resting heart rate was significantly higher in the high-endocan group (80.1 ± 11.81 bpm vs. 73.4 ± 11.14 bpm, P < 0.05); however, awake heart rate and night/day heart rate ratio was not different between high- and low-endocan groups.
de Souza et al.	2016	Cohort	Brazil	Serum	Renal transplant children between 6 and 24 months after transplant	62	12.8 ± 3.5	69.4	Serum endocan levels were significantly higher in RT children with both CKD and hypertension rather than those presenting with just one of these conditions. Moreover, patients with CKD had higher endocan levels compared to ones without CKD and hypertension (P = 0.007).
Ekiz-Bilir et al.	2019	Case-control	Turkey	Serum	Type 2 diabetes patients (normoalbuminuric and nephropathic diabetes) and healthy controls	131	56.1 ± 7.8	53.4	Patients with diabetes and nephropathy had significantly higher serum endocan levels compared to healthy controls (1175.3 [564.5–1637.5] vs. 680.77 [213.3–1433.1], P < 0.001). However, endocan levels were not significantly different between patients with diabetes and with or without nephropathy (P = 0.822).
El-Senosy et al.	2022	Case-control	Egypt	Serum	Patients with CKD (HD and non-HD) and age- and sex-matched healthy controls	90	56.2 ± 11.1	60.0	Patients with CKD and HD had significantly higher endocan levels compared to healthy controls (P < 0.05). In addition, patients who underwent HD had significantly higher levels of endocan compared to non-HD patients with CKD (519.0 [202.3–742.0] vs. 409.0 [245.3–505.3], P = 0.014).
Hureau et al.	2021	Retrospective cohort	France	Plasma	Patients undergoing intermittent hemodialysis in ICU	11	64.5 ± 8.5	54.5	Endocan concentration increases as the hemodialysis start and then decreases progressively. This initial increase can be the result of vascular stress or hemoconcentration in HD.
Kim et al.	2020	Prospective cohort	South Korea	Plasma	ESRD patients on HD	354	62.1 ± 12.7	66.4	Cardiovascular events (including ACS, stable angina, heart failure, ventricular arrhythmia, and cardiac or sudden death) were significantly higher in higher endocan groups compared to the lower-endocan group of patients with ESRD (22.7% vs. 12.9%, P = 0.016).
Kosir et al.	2019	Prospective cohort	Slovenia	Plasma	Chronic heart failure patients with or without kidney insufficiency (GFR < 60)	120	71 ± 11	64.2	Endocan levels in chronic heart failure patients was similar between CKD and non-CKD groups (3.55 [2.44–4.97] ng/ml vs. 3.26 [2.39–4.47] ng/ml, P = 0.309).
Lee et al.	2019	Retrospective cohort	South Korea	Plasma & urine	RT recipients	203	48.6 ± 11.9	62.6	Endocan levels showed considerable higher values in acute antibody-mediated rejection (ABMR) patients than in acute tubular necrosis (ATN), acute pyelonephritis (APN) and T-cell-mediated rejection (TCMR).
Malyszko et al.	2018	Case-control	Poland	Serum	RT recipients and healthy controls	85	48.4 ± 11.8	NR	Higher endocan level in RT recipients than healthy controls were found (1.70 [0.5–10.6] ng/ml. vs. 0.6 [0.2–1.2] ng/ml, P < 0.001). Moreover, the higher endocan concentration in recipients was correlated with decrease in long-term renal function after transplantation and causing grafts dysfunction, graft rejection, increased inflammatory state and infection and also hypertension.
McMillan et al.	2017	Cross sectional	United States	Plasma	Stage 5 CKD patients undergoing HD and healthy controls	140	52.9 ± 8.9	50.0	Patients with CKD undergoing HD had significantly higher endocan levels compared to healthy controls (2.18 ± 0.48 pg/ml vs. 1.81 ± 0.06 pg/ml, P = 0.017).
Oka et al.	2017	Cohort	Japan	Serum	Peritoneal dialysis patients	21	56.5 ± 12.6	61.9	Patients with rapid decrease in urine volume showed significantly higher levels of endocan compared to patients with slow decrease in urine volume (6.28 ± 4.47 ng/ml vs. 2.92 ± 1.25 ng/ml, P = 0.036).
Oltean et al.	2013	Case-control	Sweden	Plasma	Deceased brain dead (DBD) multiorgan donors and living kidney donors (LD) as healthy controls	89	50.6 ± 15.1	49.4	Significantly higher endocan levels were found in DBD than in LD (2.14 [0.65–12.36] ng/ml vs. 1.12 [0.41–6.62] ng/ml, P < 0.0001).
Pawlak et al.	2015	Case-control	Poland	Plasma	CKD patients and healthy controls	82	53.6 ± 11.0	54.7	Endocan levels were significantly higher in patients with CKD compared to healthy controls (P < 0.05). Moreover, presence of cardiovascular diseases in CKD patients was associated with higher endocan levels (1.44 [range: 0.20–5.63] ng/ml vs. 0.80 [range: 0.01–1.99] ng/ml, P < 0.05).
Perrotti et al.	2017	Prospective cohort	France	Plasma	CKD patients with and without post cardiac surgery pulmonary infection	20	77.2 ± 7.8	55.0	Plasma levels of endocan (preoperatively, 12h, and 24h post-surgery) in CKD patients with and without pulmonary infection after surgery was not different (P > 0.05). However, plasma endocan levels were significantly higher 6 hours post-surgery (24.16 ± 15.64 vs. 6.44 ± 3.16, P = 0.03).
Poon et al.	2019	Cohort	China	Serum	Peritoneal dialysis patients	193	58.8 ± 11.6	61.1	Endocan level was not independent factor for predicting mortality in peritoneal dialysis patients after adjusting for clinical confounders.
Samouilidou et al.	2018	Case-control	Greece	Serum	CKD, HD, and healthy controls	135	62.7 ± 19.3	51.1	Endocan levels were significantly higher in HD patients (146 [46 – 337] pg/ml) compared to healthy controls (81 [16 – 146] pg/ml, P < 0.001) and CKD patients without HD (86 [26 – 207] pg/ml, P < 0.001).
Su et al.	2014	Cross sectional	Taiwan	Serum	Renal transplant patients	97	43.6 ± 13.2	55.7	Endocan levels were higher in more severe CKD patients. Moreover, patients with progression of CKD had significantly higher endocan levels compared to those without progression of CKD (966.3 ± 718.2 pg/ml vs. 593.8 ± 520.5 pg/ml, P = 0.004).
Xu et al.	2022	Cross sectional	China	Plasma	Patients with hyperuricemic nephropathy (HN) and CKD stages 1,2 vs. 3,4	80	51.7 ± 10.9	96.2	Plasma endocan levels were significantly higher in patients with CKD stage 3–4 compared to patients with CKD stage 1–2 (131.33 [60.02–199.01] pg/ml vs. 81.78 [48.23–120.75] pg/ml, P = 0.034).
Yilmaz et al.	2014	Case-control	Turkey	Plasma	CKD stage 1–5 and controls	311	45.8 ± 13.5	51.0	Patients with CKD had higher endocan levels compared to non-diabetic non-CKD healthy controls (4.7 [1.9–9.4] ng/ml vs. 1.2 [1.1–1.5] ng/ml, P < 0.001).

Data are presented as mean ± standard deviation or median [interquartile range] or percentage. CKD: Chronic kidney disease; RT: Renal transplant; HD: Hemodialysis; ICU: Intensive care unit; ESRD: End-stage renal disease; ACS: Acute coronary syndrome; GFR: Glomerular filtration rate; DBD: Deceased brain dead; NR: Not reported.

**Table 2:** Quality assessment of included studies based on the Newcastle-Ottawa Scale (NOS).

Study	Selection				Comparability	Outcome		Overall Score
	Representation	Sample size	Non-Respondents	Exposure		Outcome	Statistical test	
Atis et al. (2016) [19]	*	*	*	**	-	**	*	8
Bao et al. (2018) [20]	*	*	*	**	-	**	*	8
de Souza et al. (2016) [9]	*	*	*	**	-	**	*	8
Ekiz-Bilir et al. (2019) [21]	*	*	*	**	**	**	*	10
El-Senosy et al. (2022) [22]	*	*	*	**	**	**	*	10
Hureau et al. (2021) [23]	*	*	*	**	-	**	*	8
Kim et al. (2020) [6]	*	*	*	**	-	**	*	8
Kosir et al. (2019) [11]	*	*	*	**	-	**	*	8
Lee et al. (2019) [24]	*	*	*	**	-	**	*	8
Malyszko et al. (2018) [25]	*	*	*	**	-	**	*	8
McMillan et al. (2017) [28]	*	*	*	**	-	**	*	8
Oka et al. (2017) [27]	*	*	*	**	-	**	*	8
Oltean et al. (2013) [28]	*	*	*	**	-	**	*	8
Pawlak et al. (2015) [10]	*	*	*	**	-	**	*	8
Perrotti et al. (2017) [29]	*	*	*	**	-	**	*	8
Poon et al. (2019) [30]	*	*	*	**	-	**	*	8
Samouilidou et al. (2018) [31]	*	*	*	**	-	**	*	8
Su et al. (2014) [8]	*	*	*	**	-	**	*	8
Xu et al. (2022) [32]	*	*	*	**	-	**	*	8
Yilmaz et al. (2014) [3]	*	*	*	**	-	**	*	8

### 3.3. Endocan in CKD

#### 3.3.1. Meta-analysis of Endocan levels in CKD patients and controls

**Fig 2:**
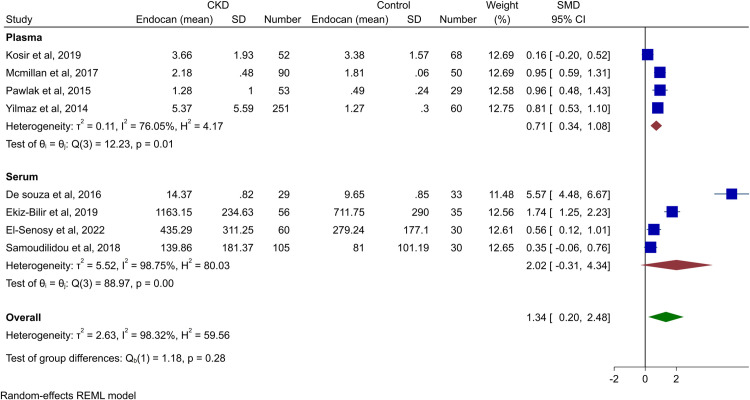
Forest plot for the meta-analysis of endocan levels in patients with CKD vs. healthy controls.

Overall, our meta-analysis included 696 CKD patients and 335 controls from eight studies [3,9–11,21,22,26,31]. The meta-analysis of these studies revealed significantly higher circulating Endocan levels in CKD patients (SMD 1.34, 95% CI 0.20 to 2.48, p-value<0.01, Fig 2). However, the heterogeneity was high (*I*^2^ = 98.32%). This trend was also seen separately in studies that reported plasma endocan (SMD 0.71, 95% CI 0.34–1.0, p-value<0.01). However, the subgroup of studies that reported serum levels did not reveal any significant difference (SMD 2.02, 95% CI -0.31 to 4.34, p-value = 0.09).

**Fig 3:**
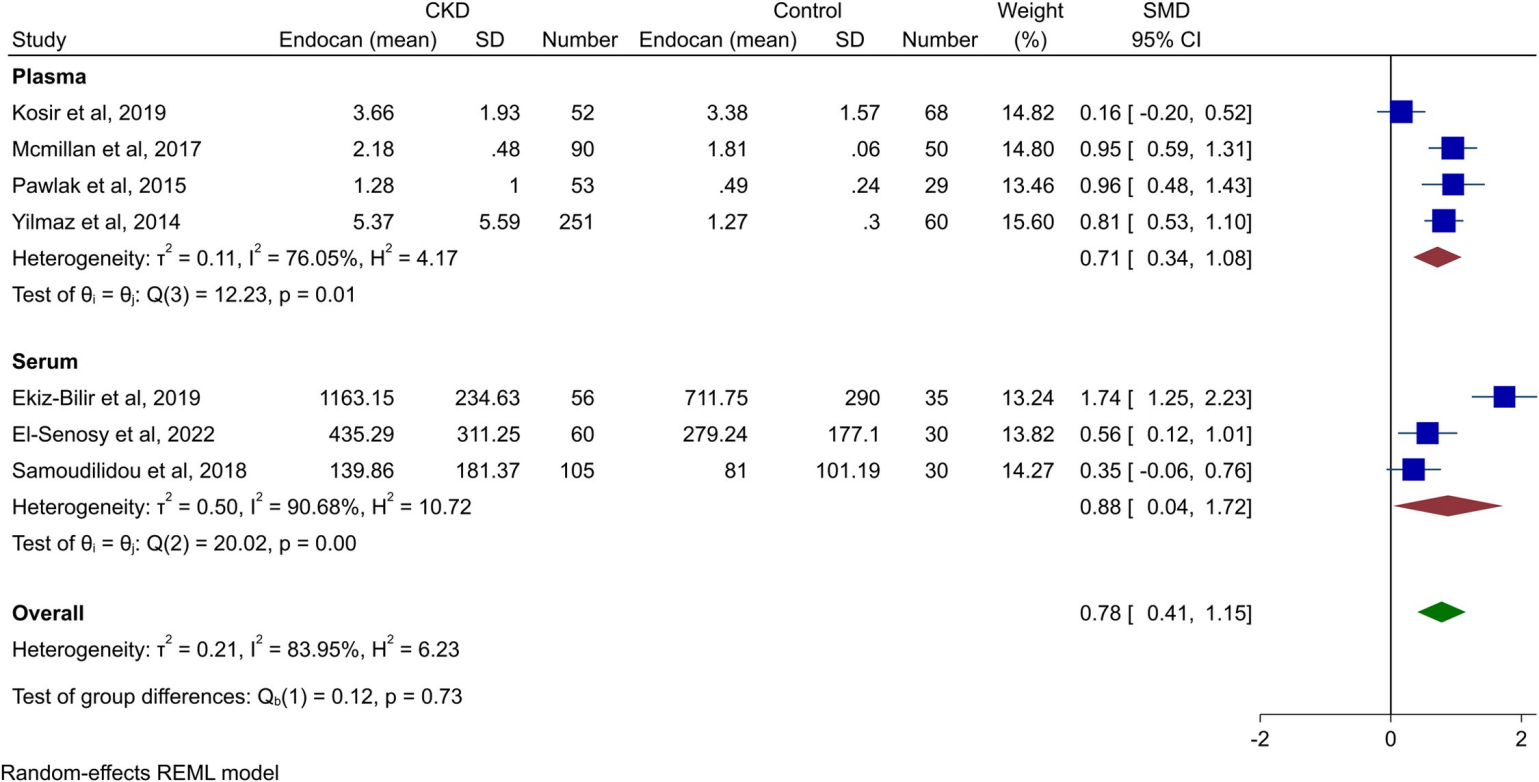
Forest plot for the meta-analysis of endocan levels in patients with CKD vs. healthy controls without outlier studies.

Any outlier study was identified and the Galbraith plot is shown in S1 Fig in S1 File. After excluding the outlier study [9], the same trend of higher endocan levels in CKD patients was seen (SMD 0.78, 95% CI 0.41–1.15, p-value<0.01), and the heterogeneity remained high (*I*^2^ = 83.95%). The forest plot for this analysis is available in Fig 3.

#### 3.3.2. Meta-regression analysis and publication bias assessment

Meta-regression was conducted to assess the source of heterogeneity and assessment of each variable’s impact on the overall pooled effect. There was a significant linear relationship between mean age and SMD (p-value<0.001). Moreover, mean age and male percentage contributed to 83.54% and 20.93% of the heterogeneity observed (Table 3). The bubble plots for these meta-regression analyses are shown in S2-S5 Figs in S1 File.

**Table 3:** Meta-regression of endocan levels in patients with CKD vs. controls.

Moderator	No. of Comparisons		Meta-regression				R^2^ Analog (proportion of variance explained)
	CKD	Control	Slope	95% CI		p-value	
**Mean Age (years)**	636	305	-0.0914	-0.1289	-0.0538	<0.001	83.54%
**Publication Year**	696	335	-0.1739	-0.6722	0.3243	0.494	0%
**Male percentage**	696	335	0.1312	-0.0171	0.2795	0.083	20.93%
**Sample Size**	696	335	-0.0075	-0.0233	0.0083	0.352	0%

CKD: Chronic kidney disease; CI: Confidence interval.

#### 3.3.3. Sensitivity analysis

The effect of each study on the overall analysis result was assessed by removing each of the studies. Our analyses showed that the removal of none of the studies could affect the overall pooled effect except for Ekiz-Bilir et al. [21]. Removal of this study changed the result from significant to insignificant (SMD 1.29, 95% CI -0.03 to 2.61, p-value = 0.06).

#### 3.3.4. Meta-analysis of Endocan levels in CKD patients without comorbidities

**Fig 4:**
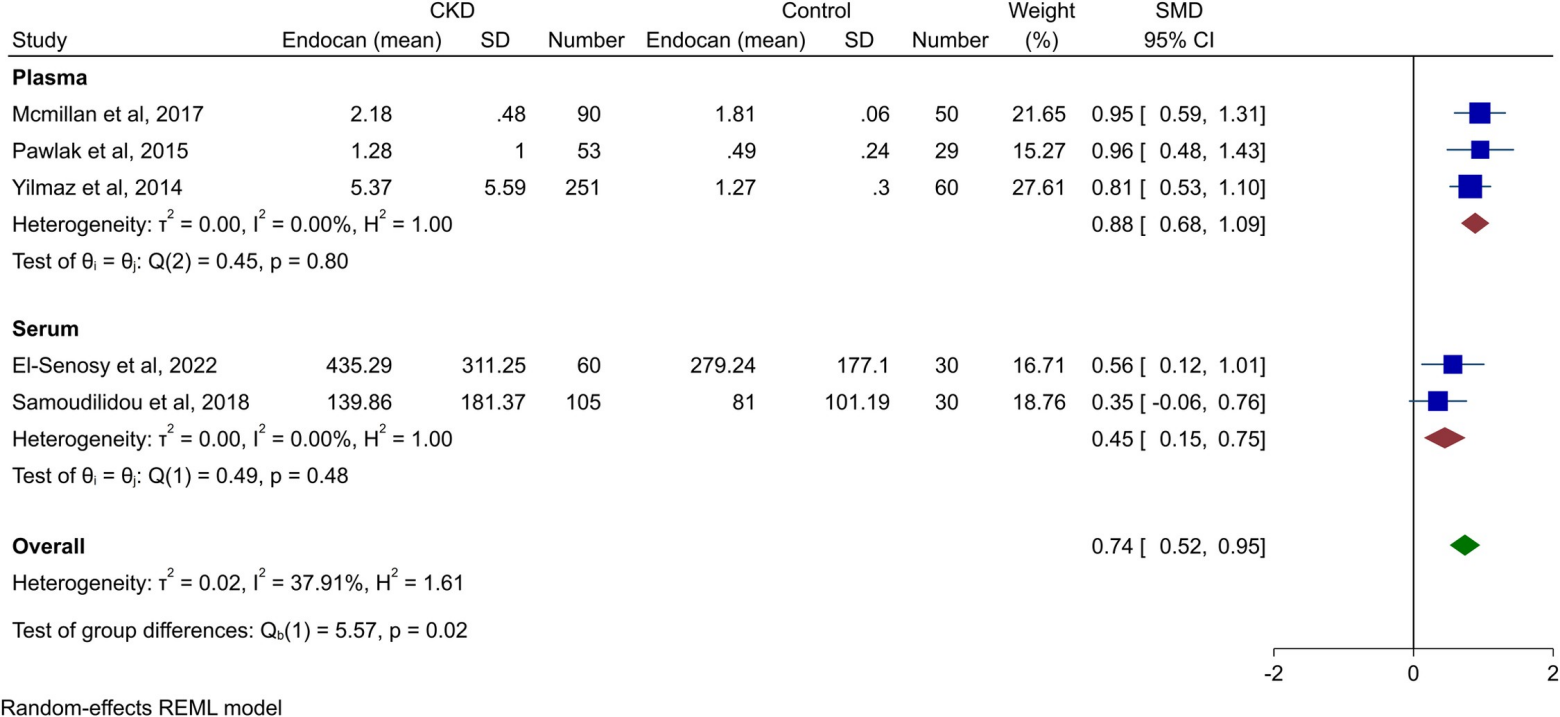
Forest plot for the meta-analysis of endocan levels in patients with CKD and without other comorbidities vs. healthy controls.

Since three of the included studies reported peripheral endocan in CKD patients that already had underlying comorbidity (chronic heart failure [11], renal transplant [9], and type 2 diabetes [21]), excluding these studies could be beneficial in elucidating the impact of CKD alone. Assessment of endocan levels in CKD patients without comorbidities showed significantly higher endocan levels in comparison to controls (SMD 0.74, 95% CI 0.52–0.95, p-value<0.01) with moderate heterogeneity (*I*^2^ = 37.91%). This was both observed in serum and plasma subgroups, as shown in Fig 4.

#### 3.3.5. Endocan in different stages of CKD

Endocan levels were assessed based on different stages of CKD in two studies [8,32]. Xu et al. [32] investigated the difference in levels of biomarkers between patients with hyperuricemic nephropathy (HN) with CKD stage 1–2 (early stage) and HN with CKD stage 3–4 (middle and late stages). Their study enrolled 80 participants. As a result, they found that plasma endocan levels were significantly higher in CKD stage 3–4 compared to CKD stage 1–2 (131.33 [60.02–199.01] pg/ml vs. 81.78 [48.23–120.75] pg/ml, p-value = 0.034). By showing that, as CKD progresses, endothelial dysfunction becomes more severe. In another cross-sectional study, Su et al [8] investigated the association of serum endocan with CKD stage in renal transplant (RT) recipients. They enrolled 97 RT recipients and stratified them by eGFR (stages 1–2, stage 3, and stages 4–5 of CKD). Endocan levels were insignificantly elevated in the late stages of CKD (p-value = 0.077). Moreover, the group with higher serum endocan (≥643.19 pg/mL) had higher rates of CKD progression during 3 months of follow-up (62%; p-value = 0.001), higher creatinine levels (1.2±0.4 vs 1.6±1.1 mg/dL; p-value = 0.029), and lower eGFR (67.8±23.8 mL/min vs. 54.4±22.0; p-value = 0.006) than those with lower serum endocan (≤643.19 pg/mL). Furthermore, linear regression analysis revealed TNF-α had a significant linear correlation with serum endocan (r = 0.286; p-value = 0.002). They suggested that endocan may reflect be used as a marker of endothelial cell injury in RT recipients and could be represented as a potential biomarker of chronic renal allograft injury (CRAI).

#### 3.3.6. Endocan in hemodialysis

The comparison of endocan levels between CKD patients on regular hemodialysis (HD) and non-dialyzed CKD patients was done in two studies [22,31]. Samouilidou et al. [31] evaluated serum endocan in CKD patients with or without HD. They found that serum endocan levels were significantly higher among HD patients in comparison with CKD without HD patients (146 [46 – 337] pg/ml vs. 86 [26 – 207] pg/ml, p-value < 0.001). Also, El-Senosy et al. [22] aimed to compare serum endocan levels in CKD without HD and CKD with HD participants and found significantly higher endocan in CKD with HD compared to CKD without HD participants (519.0 [202.3–742.0] vs. 409.0 [245.3–505.3], P = 0.014). Also, HD patients had a significantly higher percentage of subclinical atherosclerosis compared to CKD patients without HD (46.7% vs. 13.3%, p-value = 0.005). Moreover, the correlation analysis revealed that endocan concentrations were positively correlated with parathyroid hormone (PTH) levels (r = 0.5, p-value = 0.005), and carotid intima-media thickness (CIMT) (r = 0.59, p-value = 0.001). Besides, Hureau et al. [23] collected serial measurements of plasma endocan during routine HD of 11 patients. They found significant fluctuations in endocan levels during the time course of HD. Endocan concentration increased as the HD started and then decreased progressively. They suggest that the initial increase could be a consequence of vascular stress or hemoconcentration in HD.

### 3.4. Endocan as a prognostic factor in CKD

#### 3.4.1. Endocan as a prognostic factor for cardiovascular diseases

The correlation between endocan concentration in CKD patients and the risk for cardiovascular diseases, atherosclerosis, and heart rate abnormalities was assessed in four studies [3,6,20,22]. Yilmaz et al. [3] showed that endocan concentrations were associated independently with CIMT, diabetes, hypertension, and smoking, but inversely correlated with flow-mediated vasodilation. Moreover, cardiovascular events (CVE) and all-cause mortality during the time course of follow-ups (median 42 months) showed an association with endocan levels. Finally, adding endocan significantly increased the prediction capability of a model for forecasting fatal and nonfatal CVE. Kim et al. [6] evaluated the possible predictive role of endocan for cardiovascular risk in CKD patients undergoing HD. They divided patients into low-endocan and high-endocan groups. After the follow-up period (mean = 34.56 months), the high-endocan group experienced more frequent CVEs (12.9% vs. 22.7%, p = 0.016). Moreover, endocan served as an independent predictor of CVE in patients with CKD on routine HD (HR 1.95, 95% CI 1.14 to 3.32, p-value = 0.014). In another cross-sectional study, Bao et al. [20] stated that resting heart rate (HR) was significantly higher in the high-endocan patients (80.1 ± 11.81 bpm vs. 73.4 ± 11.14 bpm, p-value < 0.05). Besides, endocan acted as an independent predictor of the night/day HR ratio in CKD patients (p-value < 0.01, adjusted *R*^2^ of the model = 0.222). Also, as mentioned above, El-Senosy et al. [22] demonstrated the correlation between endocan levels and subclinical atherosclerosis in patients with CKD and HD.

#### 3.4.2. Endocan as a prognostic factor in renal transplant

To assess the correlation between peripheral endocan levels and RT patients’ blood pressure and renal function impairment, De Souza et al. [9] measured endocan concentration in 62 children undergoing renal transplantation. Their analysis revealed a positive correlation between endocan concentrations and systolic blood pressure (r = 0.416; p-value = 0.001) and pulse pressure (r = 0.412; p-value = 0.003). Besides, endocan concentrations were negatively correlated with eGFR (r = −0.388; p-value = 0.003). In a case-control study, Malyszko et al. [25] measured endocan concentration in 63 RT recipients and 22 healthy participants. As a result, significantly higher peripheral endocan levels were detected in RT recipients compared to healthy controls (1.70 [0.5–10.6] ng/ml. vs. 0.6 [0.2–1.2] ng/ml, p-value < 0.001). Moreover, endocan concentration in RT recipients was inversely correlated with eGFR (r = -0.24, p-value < 0.05), and time after transplantation (r = 0.26, p-value < 0.05), and it was positively correlated with creatinine levels (r = 0.26, p-value < 0.05). Interestingly, Atis et al. [19] evaluated vitamin D treatment in RT recipients. Endocan levels significantly decreased after treatment (from 621.5 [188–2044] pg/ml to 538 [167–2044] pg/ml, p-value = 0.001).

#### 3.4.3. Endocan as a prognostic factor in peritoneal dialysis

Oka et al. [27] collected serum and 24h urine samples in 21 individuals who underwent peritoneal dialysis (PD) and assessed the correlation between endocan levels and the participants’ clinical data. They found that endocan had a positive correlation with proteinuria level (r = 0.460, p-value = 0.036), serum creatinine level (r = 0.639, p-value = 0.0018), serum TNF-α level (r = 0.589, p-value = 0.0050), β2-microglobulin level (r = 0.479, p-value = 0.033), and PD drainage volume (r = 0.500, p-value = 0.021). In another cohort study, Poon et al. [30] explored the correlation between endocan levels and the clinical outcomes of 193 PD patients. After at least 4 years of follow-up, they demonstrated that higher endocan levels were associated with higher carotid-femoral pulse wave velocity, lower serum albumin and subjective global assessment score, and higher serum C-reactive protein. Moreover, their analysis revealed that high endocan level was an independent predictor of CVE-free survival in patients with suboptimal blood pressure control.

## 4. Discussion

To the best of our knowledge, this is the first systematic review and meta-analysis considering the association between endocan levels and CKD. The main findings include: 1) There is a progressive elevation in endocan concentration in the late stages of CKD, 2) Patients undergoing HD showed higher levels of endocan compared with both CKD and control groups, 3) Higher endocan levels correlate with increased risk of CVEs in CKD patients, and 4) RT patients showed higher endocan levels. Moreover, increasing endocan concentration was associated with transplant rejection.

CKD is a serious global health burden imposing considerable morbidity and mortality rates [21]. The persistent inflammation in distressed kidneys will cause disease progression to the more advanced stages and finally require HD or RT. Moreover, the inflammatory cascades and endothelial injury may cause graft rejection or developing renal failure. Chronic inflammation is probably present as both a source and a result of glomerular and tubulointerstitial pathology, regardless of the cause of CKD. In kidneys, endocan is highly expressed and localized in the peritubular cells of the medulla and the cortex [33,34]. Extracellular matrix (ECM) build-up and elevated inflammatory cytokines lead to tubulointerstitial fibrosis, mesangial expansion, and a later decline in GFR [35].

Endocan is secreted by vascular endothelial cells and can be detected in blood, and is a marker responsible for major processes such as cell adhesion, regulating proliferation, differentiation, and migration [25]. The role of endocan has been evaluated in several diseases [36–39]; however, our study is the first systematically evaluate its association with CKD. Mechanistically, vascular inflammation which leads to an adhesion cascade might play a role in the pathophysiology of CKD through the accumulation of leukocytes in the endothelium. This process is mediated by upregulating adhesion molecules including intracellular cell adhesion molecule-1 and vascular cell adhesion molecule-1 [40]. It has been shown elsewhere that these factors are increased in CKD [41].

Xu et al. [32] compared plasma endocan levels of 80 patients with hyperuricemic nephropathy (HN) in different stages of CKD in a cross-sectional study and showed higher endothelial inflammatory markers including endocan in CKD stages 3–4 than in those with CKD stages 1–2. Other studies also showed higher endocan concentration in HD patients compared to patients in the end stages of CKD and also with their healthy counterparts [22], emphasizing higher endocan levels in higher stages of CKD. These suggest that there might be a relationship between the CKD stage and endocan levels. Future studies assessing the CKD complications and clinical utilization of endocan in this regard are warranted.

Oka et al. [27] showed a positive correlation between baseline peripheral endocan concentration with serum creatinine, proteinuria level, serum TNF-α, and PD drainage volume. Moreover, patients with higher endocan levels and proteinuria developed a rapid decrease in their urine volume. As circulating toxins increase in CKD patients, toxin-induced endothelial injury by mediators such as TNF-α and IL-1β could cause an elevation in endocan levels [42–44]. Although many biomarkers for CKD have been proposed for diagnosing CKD so far [45,46], the correlation of endocan with creatinine can indicate that endocan can be used as a marker for the diagnosis of CKD and other kidney diseases in the future.

Vascular endothelial cells are the first site of contact between recipient and donor and are the major place of graft rejection happening by the immune system in RT recipients [24]. Despite the novel immunosuppressive regimen, graft rejection is still a substantial problem among patients. Understanding the main pathophysiology underlying rejection and the ability to earlier detection of it will provide us with profitable interventions to prevent this infelicitous hazard. Su et al. [8] showed a promising role of endocan levels as a biomarker for determining the extent of endothelial injury in RT patients and long-term outcomes. Endocan levels were higher in the acute antibody-mediated rejection (ABMR) [24].

Considering complications, cardiovascular diseases are the leading cause of mortality in CKD patients [47]. Many CKD patients die of CVEs before reaching the final stages of renal failure. It is well known that cardiac damage starts in the very early stages of CKD [3,6,22]. Moreover, the prolonged pressure and volume overload and seemingly endless vascular inflammation and oxidative stress cause cardiomyopathy and cardiac remodeling which ultimately results in heart failure [26]. Yilmaz et al. found serum endocan levels as a useful marker for estimating the survival rate of CVEs among non-dialysis CKD patients [3]. Endocan is also significantly elevated in chronic heart failure and is associated with long-term prognosis in patients with both heart failure and CKD [11]. Several experiments discovered that a high resting HR is associated with higher cardiovascular complications. Bao et al. [20] found that elevated serum endocan levels are associated with a higher night/day HR ratio in non-dialysis stage 5 CKD patients.

There were some limitations to this study. To begin with, we were unable to perform a meta-analysis between different stages of CKD and also between existing comorbidities. In addition, the other major limitation was the low small sample size of studies in some instances in meta-analysis with high heterogeneity. Third, we were unable to draw a conclusion for finding an association between renal clearance and endocan levels which highlights the need for future studies targeting this. Finally, we extracted the mean and SD levels of endocan from the median and IQR in some of the studies which although used previously in meta-analysis studies, may cause statistical errors.

## 5. Conclusion

We found significantly higher endocan in CKD patients compared to healthy controls in addition to higher circulating levels in complications of CKD. In conclusion, endocan showed promising impress in predicting cardiorenal complications among CKD patients. The higher endocan level was associated with major endothelial damage resulting in the progression of CKD, a higher probability of transplant rejection, and higher CVEs and mortality.

## Supporting information

S1 File Supplementary tables and figures. (DOCX)
